# Investigation of Factors Affecting Aerobic and Respiratory Growth in the Oxygen-Tolerant Strain *Lactobacillus casei* N87

**DOI:** 10.1371/journal.pone.0164065

**Published:** 2016-11-03

**Authors:** Rocco G. Ianniello, Teresa Zotta, Attilio Matera, Francesco Genovese, Eugenio Parente, Annamaria Ricciardi

**Affiliations:** 1 Scuola di Scienze Agrarie, Forestali, Alimentari e Ambientali, Università degli Studi della Basilicata, Potenza, Italy; 2 Istituto di Scienze dell’Alimentazione-CNR, Avellino, Italy; 3 Dipartimento di Scienze, Università degli Studi della Basilicata, Potenza, Italy; Universite Paris-Sud, FRANCE

## Abstract

Aerobic and respiratory cultivations provide benefits for some lactic acid bacteria (LAB). Growth, metabolites, enzymatic activities (lactate dehydrogenase; pyruvate and NADH oxidases, NADH peroxidase; catalase), antioxidant capability and stress tolerance of *Lactobacillus casei* N87 were evaluated in anaerobic, aerobic and respiratory (aerobiosis with heme and menaquinone supplementation) batch cultivations with different dissolved oxygen (DO) concentrations. The expression of *pox* (pyruvate oxidase) and *cydABCD* operon (cytochrome *bd* oxidase complex) was quantified by quantitative Real Time polymerase chain reaction. Respiration increased biomass production compared to anaerobiosis and unsupplemented aerobiosis, and altered the central metabolism rerouting pyruvate away from lactate accumulation. All enzymatic activities, except lactate dehydrogenase, were higher in respiratory cultures, while unsupplemented aerobiosis with 60% of DO promoted H_2_O_2_ and free radical accumulation. Respiration improved the survival to oxidative and freeze-drying stresses, while significant numbers of dead, damaged and viable but not cultivable cells were found in unsupplemented aerobic cultures (60% DO). Analysis of gene expression suggested that the activation of aerobic and respiratory pathways occurred during the exponential growth phase, and that O_2_ and hemin induced, respectively, the transcription of *pox* and *cydABCD* genes. Respiratory cultivation might be a natural strategy to improve functional and technological properties of *L*. *casei*.

## Introduction

Lactic acid bacteria (LAB) are recognized as oxygen-tolerant anaerobes, usually lacking catalase and an active electron transport (ET) chain. Although fermentative metabolism is the most frequent pathway for energy production in LAB, genomic information for several species indicates that many strains are genetically equipped to perform aerobic and respiratory growth ([1, 2]; Integrated Microbial Genomes database, IMG; http://img.jgi.doe.gov/).

The aerobic (presence of O_2_, no cofactors) pathway for energy production involves pyruvate oxidase (POX) and acetate kinase (ACK) activities, which promote the oxidation of pyruvate into acetate with production of CO_2_, H_2_O_2_ and extra ATP. The higher energy gain and pH in aerobic conditions may contribute to cell viability and robustness.

In most LAB species the activation of aerobic respiration requires exogenous supplementation of hemin or hemin and quinone (usually menaquinone). In fact, while the *pox* gene is widely distributed among LAB species, to our knowledge no strain has the complete heme biosynthesis pathway, and only few members of *Enterococcus*, *Lactococcus*, *Leuconostoc* and *Weissella* genera have the complete (ubi/mena)quinone gene complex (encoding by *menFDHBEC*, *menA*, *menG* or *UbiE* genes; [2]; IMG database). Using exogenous heme and menaquinone, a minimal respiratory chain, consisting of an electron donor (NADH dehydrogenase), a quinone electron shuttle (menaquinone) and a terminal O_2_ reductase (cytochrome *bd* oxidase) may be activated [3, 2]. Cytochrome *bd* oxidase is the only quinol oxidase in the ET chain of LAB [2], while other oxidase families (i.e. heme-copper cytochrome *c*-, ba_3_-, cbb_3_-type oxidases; alternative oxidase AOX) may be present in eukaryotes and/or prokaryotes [4]. Cytochrome *bd*-type reduces the molecular O_2_ to water, generating a proton motive force across the membrane and favouring ATP generation through F_0_F_1-_ATPase activity [5]. In prokaryotes the membrane oxidase may also promotes physiological functions (i.e. colonization of O_2_-poor environments by pathogenic and commensal bacteria, O_2_-scavenging activity, survival to stress; [4]) that may contribute to the robustness of bacterial cells. In LAB cytochrome *bd* oxidase is encoded by *cydABCD* operon and contains two structural (cytochrome *bd*-I ubiquinol oxidase subunit I, CydA; cytochrome *bd*-I ubiquinol oxidase subunit II, CydB) and two assembly (ATP-binding cassette transporters, cydC and cydD) subunits. CydCD is required for synthesis of functional cytochrome *bd* oxidase, since promote the insertion of heme group into CydAB core. The implication of iron-sulphur (Fe-S) proteins in respiratory electron transfer and proton efflux has never been demonstrated in LAB.

The aerobic and respiratory pathways have been investigated in *Lc*. *lactis* [1, 6, 2], *Lactobacillus plantarum* [7–10], and more recently [11–13] in some members of *L*. *casei* group. The O_2_-tolerant and respirative phenotypes had enhanced technological (greater production of biomass and aroma compounds) and stress response (robustness, increased survival and antioxidant capability) properties. Several of these studies, however, were carried out in shaken flasks without pH and O_2_ control, often comparing anaerobic growth with only one of aerated conditions (i.e. anaerobiosis *vs* unsupplemented aerobiosis or anaerobiosis *vs* respiration). Moreover, although factors inducing the expression of the *pox* gene have been investigated [14–15], the information on the transcription of *cydABCD* operon are still scant.

In this work, we have evaluated the effect of anaerobic, aerobic (O_2_) and respiratory (O_2_, heme and menaquinone) growth, as well as of dissolved oxygen concentration (30% or 60% DO) on the biomass yield, metabolites, activities of O_2_-related enzymes, antioxidant capability and stress tolerance of respiration-competent strain *L*. *casei* N87. The expression of *pox* and *cydABCD* operon was investigated for the first time in the different growth conditions and phases to elucidate the possible factors (growth phase, O_2_ parameters, heme supplementation) affecting the activation of aerobic and respiratory pathways in *L*. *casei*.

## Materials and Methods

### Strain and culture conditions

*Lactobacillus casei* N87 [12, 16] was used in this study. The strain was maintained as freeze-dried stock in reconstituted 11% (w/v) Skim Milk containing 0.1% (w/v) ascorbic acid, in the culture collection of the Laboratory of Industrial Microbiology, Università degli Studi della Basilicata, and was routinely propagated in Weissella Medium Broth, pH 6.8 (WMB; [17]), for 16 h at 37°C.

### Fermentation conditions

The growth of *L*. *casei* N87 was evaluated for 24 h at 37°C in modified WMB (10 g/l glucose, without sodium acetate and sodium citrate; mWMB) in batch cultivations carried out under anaerobic (nitrogen flow at 0.1 vol/vol/min; AN), aerobic (30% or 60% dissolved oxygen concentration, DO; AE30 or AE60, respectively) and respiratory (supplementation of mWMB with 2.5 μg/ml hemin and 1 μg/ml menaquinone; with 30% or 60% of DO; RS30 or RS60, respectively) conditions.

Bioreactors (3 l working volume; Applikon, Schiedam, the Netherlands) were inoculated (1% v/v) with an overnight (16 h, 37°C) WMB anaerobic pre-culture, washed twice in 20 mM potassium phosphate buffer pH 7 (PB7). DO was measured using a polarographic electrode (Applisens, Applikon) and was automatically controlled (*ez*Control controller, Applikon; set point 30% or 60%) by varying the stirrer speed (impeller speed from 200 to 800 rpm; two Rushton turbines, 45 mm diameter) and the opening (from 0% to 100%) of air flow valve (1 vol/vol/min maximum air flow). pH was controlled (pH 6.5) by automatic addition of sterile 4 eq/l NaOH, while foaming was controlled by automatic addition of a sterile 5% (v/v) Antifoam A solution.

Two independent cultivations were carried out for each growth condition (AN, AE30, AE60, RS30, RS60).

### Kinetics of growth during batch cultivation

Samples were aseptically withdrawn every hour for the measurement of absorbance at 650 nm (A_650_; SmartSpec^™^130 Plus; Bio-Rad Laboratories) and, in exponential and stationary growth phases, a standard curve relating A_650_ and cell dry weight (CDW; washed biomass dried at 105°C for 24 h) was used to estimate the biomass concentration. Growth kinetics was modeled using the dynamic model of Baranyi and Roberts [18] using DMFit v.2.0 program [19].

### Chemical and biochemical analyses

Consumption of O_2_ (resazurin method; [11]), residual glucose and production of lactate, acetate, citrate (enzymatic kits; R-Biopharm AG, Darmstadt, Germany) and H_2_O_2_ in the supernatants [12], were measured after 5 h (exponential phase), 7 h (late exponential phase), 9 h (early stationary phase) and 24 h (late stationary phase). Two technical replicates were carried out for all analyses.

### Activities of enzymes related to the aerobic growth and oxidative stress

The activities of pyruvate oxidase (POX), NADH-dependent oxidase (NOX), NADH-dependent peroxidase (NPR), lactate dehydrogenase (LDH) and catalase (CAT) were measured after 5, 7, 9 and 24 h of growth in cell free extracts (mechanical lysis with FastPrep-24 Instrument, MP Biomedicals, California, USA; 5 cycles of 60 s at speed 6.0), according to Zotta et al [12]. Two technical replicates were used for each experiment.

### Radical scavenging activity and survival to oxidative stress and freeze-drying

Radical scavenging activity and stress tolerance was evaluated on exponential (E) and late stationary (S; 24 h of growth) phase cells. Cell suspensions were standardized to A_650_ = 1.0 before the assay. The capability to remove 1,1-diphenyl-2-picrylhydrazil (DPPH)- and hydroxyl-radicals was measured on whole cells using the methods described by Wang et al [20]. Tolerance of oxidative stress was tested by exposing the cell suspensions (washed twice and re-suspended in PB7 to a final A_650_ = 1.0) to H_2_O_2_ (50 mM), pyrogallol (300 mM) or menadione (5 mM) for 30 min at 37°C. Different fractions of survivors were evaluated by pour plating using a variety of media. Total survivors were enumerated using WMB containing 1.2% agar bacteriological, pH 6.8, supplemented 0.05% w/v of cysteine (WMAC). Healthy cells were enumerated in WMA pH 6.8, without cysteine (WMA68). Undamaged cells were enumerated on WMA pH 5.5 without cysteine (WMA55). All media were incubated for 48 h at 37°C, in anaerobiosis. As a result, sub-lethally damaged cells were estimated using the difference between counts on WMA55 and WMA65, while viable but non-cultivable (VBNC) cells, which required the presence of a scavenger of reactive oxygen species, were estimated using counts on WMAC and WMA65. Dead cells were estimated from counts on WMAC before and after the stress treatment.

Survival to freeze-drying was measured on the cell suspensions (A_650_ = 1.0) re-suspended in Skim Milk containing 0.1% w/v ascorbic acid and freeze-dried at -50°C, 0.1 hPa for 24 h (Heto Drywinner 3 Benchtop freeze-drier). The number of healthy, damaged and VBNC cells, after 60 and 90 days of storage at -20°C, was estimated as described before. Two technical replicates were carried out for each treatment.

### Expression of pyruvate oxidase and cytochrome oxidase genes

#### Genome sequencing and primer design

DNA for genome sequencing was isolated from the stationary phase culture of *L*. *casei* N87 by using the GeneElute Bacterial Genomic DNA Kit (Sigma-Aldrich). The quality and quantity of DNA was assessed using both agarose gel electrophoresis and a NanoDrop^®^ 1000c spectrophotometer (Thermo Scientific, Wilmington, DE). The whole genome sequencing (WGS) of *L*. *casei* N87 was performed using an Illumina HiSeq 1000 platform (Centre of Functional Genomics, Department of Science and Technology, University of Verona, Italy) and the WGS shotgun project was deposited at DDBJ/EMBL/GenBank with the accession no. LCUN00000000 [21]. Sequences encoding for pyruvate oxidase (*pox*), for the subunit I of cytochrome D ubiquinol oxidase (*cydA*), for the subunit II of cytochrome D ubiquinol oxidase (*cydB*), for the ABC transporters of cytochrome D ubiquinol oxidase (*cydC* and *cydD*), for the glyceraldehyde-3-phosphate dehydrogenase (*gapdh*), for the elongation factor Tu (*tuf*) and for the subunit B of DNA gyrase (*gyrB*) were used as template for primer design (Primer Express software 3.0, Applied Biosystems, Concord, Ontario, Canada; S1 Table).

#### RNA extraction and synthesis of cDNA

RNA was isolated from all cultures after 5, 7 and 9 h of incubation using the ZR Fungal/Bacterial RNA kit (Zymo Research, Irvine, CA, US). An on-column DNA-digestion step (DNase I RNase-free; Invitrogen^™^, Burlington, Ontario, Canada) was added to the RNA-kit protocol. RNA samples were quantified (NanoDrop^®^ 1000c spectrophotometer) and used as templates for the synthesis of complementary DNA (cDNA).

One μg of RNA was mixed (final volume of 13 μl) with 100 ng of random primers (Promega, Madison, WI, USA), 10 mM of dNTP mix (Invitrogen) and nuclease-free water (Ambion, Streetsville, Ontario, Canada) and incubated at 65°C for 5 min. After cooling on ice (at least 1 min), 4 μl of 5x first strand buffer, 100 mM of DTT, 40 units/μl of RNase OUT^™^ Recombinant RNase Inhibitor (Invitrogen) and 200 units/μl of SuperScript^™^ III RT (Invitrogen) were added to the mixture and incubated for 10 min at 25°C, 50 min at 50°C and 15 min at 70°C. cDNA samples were stored at -20°C until the use.

#### Selection of housekeeping genes and quantification of pox and cydABCD genes by quantitative Real Time PCR (qRT-PCR)

Sequence encoding for *gapdh*, *gyrB* and *tuf* were used to select the housekeeping genes. The expression of *gapdh*, *gyrB*, *tuf*, *pox* and *cydA*, *cydB*, *cydC*, *cydD* (following indicated as *cydABCD*) was quantified in all growth conditions (AN, AE30, AE60, RS30, RS60) and times of incubation (5, 7 and 9 h) as described below.

qRT-PCR was performed in a StepOne^™^ real-time PCR instrument (Applied Biosystems, Concord, Ontario, Canada), using a SYBR Green master mix (Qiagen, Toronto, Ontario, Canada). The amplification program was: 1 cycle at 95°C for 5 min, 40 cycles at 95°C for 30 s, and a final step at 60°C for 30 min.

The coefficient of variation (cv) of the threshold cycle (Ct) values of *gapdh*, *gyrB* and *tuf* was calculated to select the appropriate housekeeping gene, while the relative expression of *pox* and *cydABCD* was estimated according to the ΔΔC_t_ method [22], using *gapdh* as selected reference gene. The anaerobic exponential phase (5 h) was used as reference growth condition, while the reaction mixtures without cDNA template were used as negative controls.

Two technical replicates of the gene expression analysis were carried out for each growth experiment.

#### Reagents, culture media and ingredients

Unless otherwise stated all reagents were obtained from Sigma-Aldrich (Milan, Italy), while culture media and ingredients were obtained from Oxoid Ltd. (Basingstoke, Hampshire, UK).

### Statistical analysis

All statistical analyses (analysis of variance, multiple mean comparison Tukey’s HSD test, correlation) and graphs were performed using Systat 13.0 for Windows (Systat Software Inc., San Jose, CA, USA).

## Results

### Kinetics of growth, production of metabolites and oxygen uptake

Parameters of anaerobic, aerobic and respiratory growth of *L*. *casei* N87 are shown in Table 1.

**Table 1:** Estimated values for maximum specific growth rate (μ_max_), duration of the lag phase (λ) and maximum biomass concentration (X_max_, cell dry weight) for the kinetics of growth in anaerobic, aerobic and respiratory batch cultivations of *Lactobacillus casei* N87 in WMB. Mean values ± standard errors of 4 replicates (two biological replicates and two technical replicates of each biological experiment) are shown.

Label	DO (%)^a^	Suppl ^b^	μ_max_ (h^-1^)	lag (h)	X_max_ (g/l)	R^2^
AN	0	no	0.64±0.01	0.37±0.01	4.06±0.02	0.996±0.01
AE30	30	no	0.65±0.01	0.36±0.03	3.15±0.07*	0.997±0.01
AE60	60	no	0.48±0.00*	0.36±0.02	2.56±0.02*	0.996±0.00
RS30	30	H+M	0.65±0.01	0.37±0.01	4.24±0.01	0.996±0.01
RS60	60	H+M	0.68±0.01	0.35±0.02	5.28±0.07*	0.998±0.01

^**a**^ dissolved oxygen concentration during fermentation; RS30, respiratory growth, 30% DO, supplementation with 2.5 μg/ml hemin and 1 μg/ml menaquinone; RS60, respiratory growth, 60% DO,.

^**b**^ supplementation with μg/ml hemin (H) and 1 μg/ml menaquinone (M)

*, indicates significant differences (Tukey**’**s HSD, *p*<0.01) of aerobic (AE30, AE60) or respiratory (RS30, RS60) cultivations compared to anaerobic (AN) growth.

The D-model of Baranyi and Roberts [19] (S1 Fig) provided an excellent fit for all cultivations (R^2^ ranging from 0.996 to 0.998; Table 1). Growth at 60% of DO without supplementation significantly (Tukey’s HSD, *p*<0.01) reduced the maximum specific growth rate (μ_max_) compared to anaerobic growth, while μ_max_ values in the other aerated conditions (aerobiosis with 30% DO, respiration with 30% or 60% DO) were not significantly different from those obtained in anaerobiosis. Maximum biomass concentration (X, g/l) was significantly (*p*<0.01) increased during respiratory growth with 30% and 60% DO, and strongly reduced in unsupplemented aerated conditions with both 30% and 60% DO. Evidence for autolysis, with decrease in biomass concentration during the stationary phase, was found in anaerobic and respiratory (with 60% DO) cultivations.

Lactic acid was by far the most abundant product (Y_P/S_ = 98%) at the end of anaerobic cultivation, while low amounts of acetic acid were measured in the supernatants of respiratory growing cultures at all sampling points and only at the end of the stationary phase for aerated unsupplemented cultures (Table 2). Lactic acid and biomass yields in the exponential phases (E, LE) were significantly lower and higher, respectively, than those typically found in homofermentative lactic acid bacteria, even in anaerobic cultivation. The percentage of estimated pyruvate rerouted away from lactate and available for the conversion in other products (e.g. acetic acid) was higher in aerobic and respiratory cultivations. Citrate was never detected.

**Table 2:** Consumption of glucose, production of metabolites and O_2_ uptake during anaerobic, aerobic and respiratory growth of *Lactobacillus casei* N87. Mean values ± standard errors of 4 replicates (two biological replicates and two technical replicates of each biological experiment) are shown.

Label	DO (%) ^a^	Suppl ^b^	Growth phase ^c^	Biomass production (g/l) ^d^	Average biomass yield (g/g) ^e^	Average lactate yield (g/g) ^f^	Average acetate yield (g/g) ^g^	% pyr ^h^	H_2_O_2_ (μM) ^i^	O_2_ uptake (min) ^l^
AN	0	no	5 –(E)	1.07±0.02	0.31±0.02	0.59±0.00	-	40.96±0.16	-	≥ 180
			7 –(LE)	2.72±0.05	0.46±0.00	0.74±0.02	-	16.96±0.08	-	≥ 180
			9 –(ES)	4.06±0.02	0.39±0.00	0.85±0.00	-	15.04±0.05	-	≥ 180
			24 –(LS)	3.42±0.03	0.32±0.00	0.98±0.01	-	2.22±0.52	-	≥ 180
AE30	30	no	5 –(E)	0.95±0.01	0.21±0.00	0.58±0.00	-	42.01±0.06	-	88±3
			7 –(LE)	2.11±0.06	0.23±0.00	0.62±0.00	-	38.09±0.13	-	118±4
			9 –(ES)	3.04±0.01	0.29±0.00	0.66±0.00	-	33.81±0.16	24.2±1.07	150±3
			24 –(LS)	3.15±0.07	0.30±0.00	0.75±0.01	0.01±0.00	25.02±0.22	32.1±0.71	≥ 180
AE60	60	no	5 –(E)	0.45±0.01	0.17±0.03	0.55±0.01	-	45.45±0.56	-	≥ 180
			7 –(LE)	0.94±0.02	0.21±0.00	0.66±0.03	-	34.01±0.18	56.9±2.50	125±4
			9 –(ES)	1.96±0.01	0.24±0.00	0.72±0.00	-	28.04±0.21	75.5±0.53	161±4
			24 –(LS)	2.56±0.02	0.25±0.00	0.82±0.00	0.01±0.00	18.21±0.21	84.6±1.25	≥ 180
RS30	30	H+M	5 –(E)	1.13±0.06	0.47±0.03	0.58±0.01	0.05±0.00	41.58±0.88	-	80±4
			7 –(LE)	2.64±0.02	0.54±0.02	0.61±0.04	0.07±0.00	38.98±0.22	-	64±4
			9 –(ES)	3.90±0.04	0.51±0.00	0.67±0.01	0.08±0.00	32.75±0.55	-	113±4
			24 –(LS)	4.24±0.01	0.45±0.00	0.66±0.01	0.11±0.00	33.84±0.30	13.3±0.71	≥ 180
RS60	60	H+M	5 –(E)	1.18±0.03	0.34±0.01	0.67±0.01	0.07±0.00	32.87±0.52	-	73±2
			7 –(LE)	3.40±0.01	0.69±0.00	0.65±0.01	0.08±0.00	34.54±0.18	-	54±3
			9 –(ES)	5.28±0.07	0.58±0.00	0.64±0.03	0.10±0.10	35.51±0.16	-	103±5
			24 –(LS)	4.76±0.01	0.45±0.00	0.62±0.00	0.23±0.00	37.59±0.07	11.4±053	≥ 180

^**a**^ dissolved oxygen concentration during different growth conditions (AN, anaerobiosis; AE, aerobiosis; RS, respiration, i.e. aerobiosis with hemin and menaquinone).

^**b**^ supplementation with 2.5 μg/ml hemin (H) and 1 μg/ml menaquinone (M).

^**c**^ Growth phase: E, exponential growth phase (5 h of incubation); LE, late exponential growth phase (7 h of incubation); ES, early stationary growth phase (9 h of incubation); LS, late stationary growth phase (24 h of incubation).

^**d**^ Biomass production: biomass production (X-X_0_), g/l.

^**e**^ Average growth yield: calculated as **(**X-X_0_)/(S_0_-S), i.e. biomass production **(**X-X_0_) g/l, relative to consumed glucose (S_0_-S), g/l.

^**f**^ Average lactate yield: production of DL-lactic acid (P-P_0_), g/l, relative to consumed glucose (S_0_-S), g/l.

^**g**^ Average acetate yield: production of acetic acid (A-A_0_), g/l, relative to consumed glucose (S_0_-S), g/l.

^**h**^ % pyr: percentage of estimated pyruvate rerouted away from lactate and available for conversion in other products; calculated as the % of estimated mmol of pyruvate not converted into mmol of lactate.

^**i**^ H_2_O_2_: production of H_2_O_2_ (μM).

^**l**^ O_2_ uptake: Consumption of oxygen expressed as time (min) of resazurin discoloration. The values ≥ 180 indicate no O_2_ consumption.

Since the growth parameters and the metabolites measured in AE30, AE60, RS30 and RS60 were significant different (Tukey’s HSD, *p*<0.01) from those detected in AN cultivation, at the same incubation time, no letters or symbols were used in the Table 2.

The anaerobic growing cells were unable to consume O_2_, while the highest capability of O_2_ uptake was found in the late exponential cultures grown under respiratory conditions (Table 2). The stationary phase cells of *L*. *casei* N87 never reduced the redox indicator resazurin, regardless of the growth condition.

### Activities of enzymes related to the aerobic growth and oxidative stress

**Fig 1:**
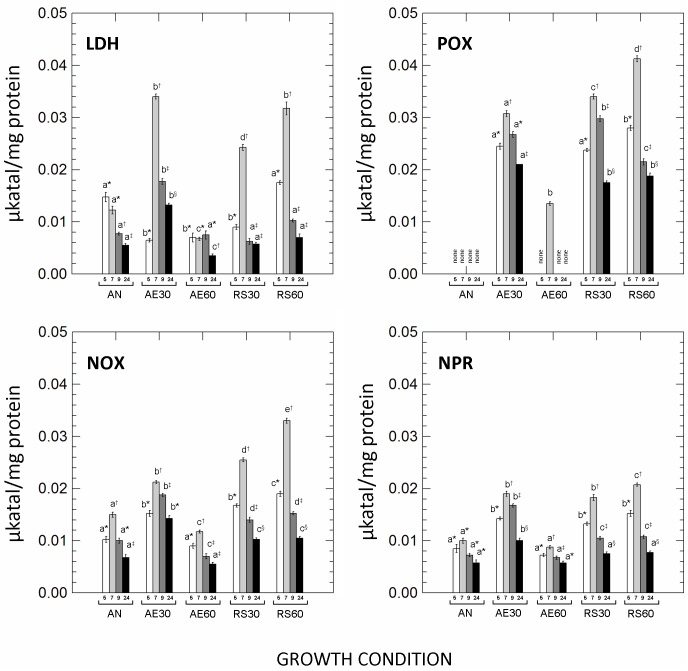
Lactate dehydrogenase (LDH), pyruvate oxidase (POX), NADH-dependent oxidase (NOX) and NADH-dependent peroxidase (NPR) activity in *Lactobacillus casei* N87 cultivated in different conditions. AN: anaerobic growth; AE30: aerobic growth with 30% dissolved oxygen, DO; AE60: aerobic growth with 60% DO; RS30: respiratory growth (supplementation with 2.5 μg/ml hemin and 1 μg/ml menaquinone) with 30% DO; RS60: respiratory growth (supplementation with 2.5 μg/ml hemin and 1 μg/ml menaquinone) with 60% DO. White bars: cultures after 5 h of incubation; light grey bars: cultures after 7 h of incubation; grey bars: cultures after 9 h of incubation; black bars: cultures after 24 h of incubation. Lowercase letters on the bars indicate significant differences (Tukey’s HSD, *p*<0.01) in enzymatic activity among different growth conditions (AN, AE30, AE60, RS30, RS60) at the same time of incubation. Symbols (*, †, ‡, §) indicate significant differences (*p*<0.01) in enzymatic activity within the same growth condition at different time of incubation.

The activities of lactate dehydrogenase (LDH), pyruvate oxidase (POX), NADH-dependent oxidase (NOX) and NADH-dependent peroxidase (NPR) are shown in Fig 1. The activity of LDH in anaerobic growing cells was highest in exponential growth phase and decreased with the time of cultivation. On the contrary, in cells grown in both respiratory conditions (30% and 60% DO) and in unsupplemented aerated cultures with 30% of DO the levels of LDH were higher in the late exponential growth phase (7 h of incubation). LDH activity was significantly (*p*<0.01) impaired during aerobic growth carried out with 60% of DO. POX activity was not detected in anaerobic cultures, but was significantly higher in lower-aerated (AE30) and respiratory (30% and 60% DO) cells of *L*. *casei* N87. The high levels (60%) of DO in unsupplemented aerated cultures dramatically reduced the POX activity. The profile of NOX and NPR activity was similar in all growth conditions, with the highest levels in the late exponential phase (7 h) and the lowest in late stationary cells (24 h). The activities of both flavin-dependent enzymes, however, were greatest in lower-aerated (30% DO) and respiratory (30% and 60% DO) cultivations.

**Fig 2:**
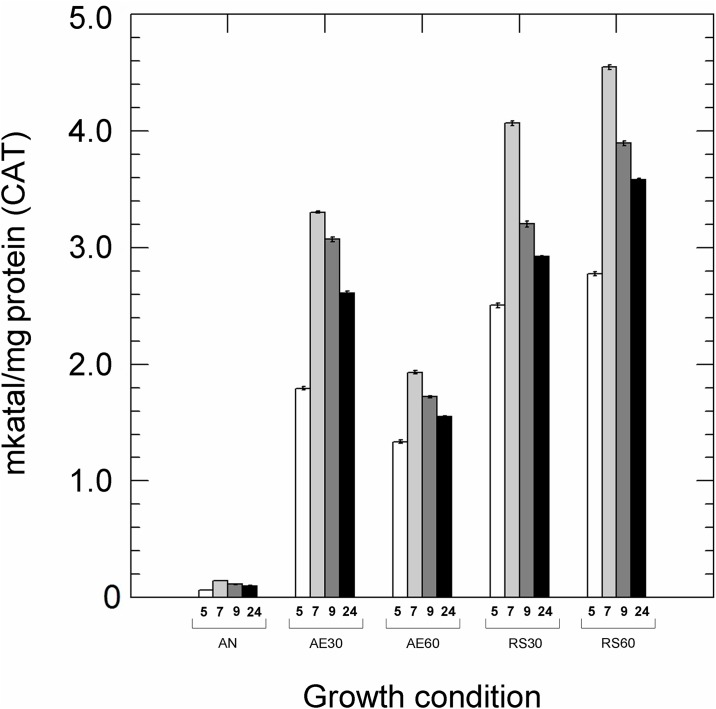
Total catalase activity in *Lactobacillus casei* N87 cultivated in different conditions. AN: anaerobic growth; AE30: aerobic growth with 30% dissolved oxygen, DO; AE60: aerobic growth with 60% DO; RS30: respiratory growth (supplementation with 2.5 μg/ml hemin and 1 μg/ml menaquinone) with 30% DO; RS60: respiratory growth (supplementation with 2.5 μg/ml hemin and 1 μg/ml menaquinone) with 60% DO. White bars: cultures after 5 h of incubation; light grey bars: cultures after 7 h of incubation; grey bars: cultures after 9 h of incubation; black bars: cultures after 24 h of incubation. The levels of catalase activity were significant different (Tukey’s HSD, *p*<0.01) in all growth conditions.

Catalase activity was significantly different (*p*<0.01) in all type of cultivations and times of incubation (Fig 2). As for others enzymes, the highest values were measured in the late exponential phases (7 h) and respiratory growing cells, while anaerobiosis and aerobiosis with high O_2_ levels significantly impaired the catalase activity. Measurable amounts of H_2_O_2_ were clearly produced by *L*. *casei* N87 in aerated conditions with high 60% DO, when the lowest NPR and catalase activities were measured (Table 2).

### Expression of genes encoding for pyruvate oxidase and cytochrome oxidase

The expression of *pox* (encoding for pyruvate oxidase, the key enzyme of aerobic metabolism) and *cydABCD* operon (encoding for cytochrome oxidase complex in respiratory chain) was evaluated by qRT-PCR. The genes encoding for the glyceraldehyde-3-phosphate dehydrogenase (*gapdh*), for the elongation factor Tu (*tuf*) and for the subunit B of DNA gyrase (*gyrB*) were used to select the suitable housekeeping gene for qRT-PCR. The coefficient of variation (cv) of the threshold cycle (Ct) values of *gapdh*, *gyrB* and *tuf*, calculated within all growth conditions and times of incubation, ranged between 0.021 and 0.027, and *gapdh* (cv = 0.021) was used as reference gene. The relative expression of *pox* and *cydABCD* operon was calculated using the exponential anaerobic growth (5 h) as reference condition (Table 3).

**Table 3:** Relative gene expression of pyruvate oxidase (*pox*) and cytochrome oxidase complex (*cydABCD*) of *Lactobacillus casei* N87. Mean values ± standard error of 4 replicates (two biological replicates and two technical replicates of each biological experiment) are shown.

Gene ^a^	Growth phase ^b^	Relative gene expression in the different growth conditions ^c^				
		Anaerobiosis	Aerobiosis with 30% DO	Aerobiosis with 60% DO	Respiration with 30% DO	Respiration with 60% DO
*pox*	5 –(E)	1.00 ± 0.00 ^a**†**^	1.32 ± 0.19 ^a**†**^	1.27 ± 0.16 ^a**†**^	1.43 ± 0.09 ^a**†**^	1.81 ± 0.22 ^b**†**^
	7 –(LE)	1.02 ± 0.14 ^a**†**^	2.50 ± 0.17 ^b §^	1.44 ± 0.08 ^a**†**^	3.22 ± 0.09 ^c §^	3.50 ± 0.11 ^d §^
	9 –(ES)	1.00 ± 0.06 ^**A †**^	1.49 ± 0.13 ^**A †**^	1.06 ± 0.26 ^**A †**^	2.01 ± 0.26 ^**B ‡**^	2.11 ± 0.15 ^**B †**^
*cydA*	5 –(E)	1.00 ± 0.00 ^a**†**^	1.18 ± 0.02 ^a**†**^	0.96 ± 0.07 ^a**†**^	2.96 ± 0.24 ^b**†**^	2.45 ± 0.13 ^b**†**^
	7 –(LE)	1.05 ± 0.12 ^a**†**^	1.34 ± 0.10 ^a**†**^	1.18 ± 0.05 ^a**†**^	3.44 ± 0.12 ^b §^	4.78 ± 0.39 ^c §^
	9 –(ES)	1.10 ± 0.10 ^**A †**^	1.22 ± 0.12 ^**A †**^	1.05 ± 0.13 ^**A †**^	2.17 ± 0.19 ^**B †**^	3.38 ± 0.20 ^**C ‡**^
*cydB*	5 –(E)	1.00 ± 0.00 ^a**†**^	1.27 ± 0.06 ^a**†**^	0.81 ± 0.25 ^a**†**^	2.87 ± 0.15 ^b**†**^	2.32 ± 0.10 ^b**†**^
	7 –(LE)	1.09 ± 0.04 ^a**†**^	1.54 ± 0.29 ^a**†**^	1.13 ± 0.18 ^a**†**^	3.50 ± 0.06 ^b §^	4.74 ± 0.21 ^c §^
	9 –(ES)	0.84 ± 0.03 ^**A** §^	1.19 ± 0.05 ^**A †**^	1.07 ± 0.14 ^**A †**^	2.27 ± 0.26 ^**B †**^	3.43 ± 0.16 ^**C ‡**^
*cydC*	5 –(E)	1.00 ± 0.00 ^a**†**^	1.22 ± 0.20 ^a**†**^	0.70 ± 0.10 ^a**†**^	1.95 ± 0.20 ^b**†**^	2.24 ± 0.06 ^c**†**^
	7 –(LE)	1.03 ± 0.03 ^a**†**^	1.23 ± 0.18 ^a**†**^	1.17 ± 0.03 ^a §^	2.45 ± 0.18 ^b §^	4.45 ± 0.31 ^c §^
	9 –(ES)	1.03 ± 0.14 ^**A †**^	1.10 ± 0.12 ^**A †**^	1.02 ± 0.05 ^**A** §^	1.80 ± 0.08 ^**B †**^	3.35 ± 0.43 ^**C ‡**^
*cydD*	5 –(E)	1.00 ± 0.00 ^a**†**^	1.16 ± 0.16 ^a**†**^	0.69 ± 0.11 ^a**†**^	2.02 ± 0.08 ^b**†**^	2.44 ± 0.15 ^c**†**^
	7 –(LE)	0.98 ± 0.07 ^a**†**^	1.12 ± 0.04 ^a**†**^	1.16 ± 0.18 ^a §^	2.40 ± 0.07 ^b §^	4.41 ± 0.19 ^c §^
	9 –(ES)	1.11 ± 0.04 ^**A †**^	1.11 ± 0.05 ^**A †**^	1.08 ± 0.06 ^**A** §^	1.88 ± 0.13 ^**B †**^	3.27 ± 0.10 ^**C ‡**^

^**a**^ Gene**:** *pox*, pyruvate oxidase; *cydA*, cytochrome bd-type quinol oxidase subunit I; *cydB*, cytochrome bd-type quinol oxidase, subunit II; *cydC*, cytochrome bd biosynthesis ABC-type transporter cydC; *cydD*, cytochrome bd biosynthesis ABC-type transporter cydD.

^**b**^ Growth phase: E, exponential growth phase (5 h of incubation); LE, late exponential growth phase (7 h of incubation); ES, early stationary growth phase (9 h of incubation).

^**c**^ Relative gene expression: relative gene expression was calculated using *gapdh* (encoding for glyceraldehyde-3-phosphate dehydrogenase) as housekeeping gene and the exponential anaerobic growth as reference condition.

Lowercase letters indicate significant differences (Tukey’s HSD, *p*<0.01) in relative gene expression among different growth conditions after 5 h of incubation; italic lowercase letters indicate significant differences (*p*<0.01) in relative gene expression among different growth conditions after 7 h of incubation; capital letters indicate significant differences (*p*<0.01) in relative gene expression among different growth conditions after 9 h of incubation; symbols (†, ‡, §) indicate significant differences (*p*<0.01) in relative gene expression among the growth phases within the same growth condition.

The relative expression of *pox* gene was significantly higher in respiratory (with 30% and 60% DO) and in lower-aerated (30% DO) cultures. The highest values of gene expression were measured in the late exponential phase cells (7 h), and a good correlation (Pearson’s *r* correlation coefficient = 0.836, *p*<0.01) between POX activity and its relative gene expression (S2 Fig) was found also within the others times of incubation.

The genes belonging to *cydABCD* operon were expressed only in respiratory conditions, suggesting that the presence of hemin was the main factor affecting the *cydABCD* transcription. The highest levels of *cydABCD* expression were found in respiratory cultures cultivated with 60% DO, even if significant values were also measured when lower (30%) O_2_ concentrations were supplied.

For both respiratory conditions (30% and 60% DO) the maximum expression of *cydABCD* was found in the late exponential phases (7 h). The expressions of *cydC* and *cydD*, encoding for the ABC transporters (cytochrome *bd* oxidase assembly) were slightly lower than those of *cydA* and *cydB*, encoding for the structural subunits of the respiratory enzyme.

### Antioxidant capability and stress tolerance

**Fig 3:**
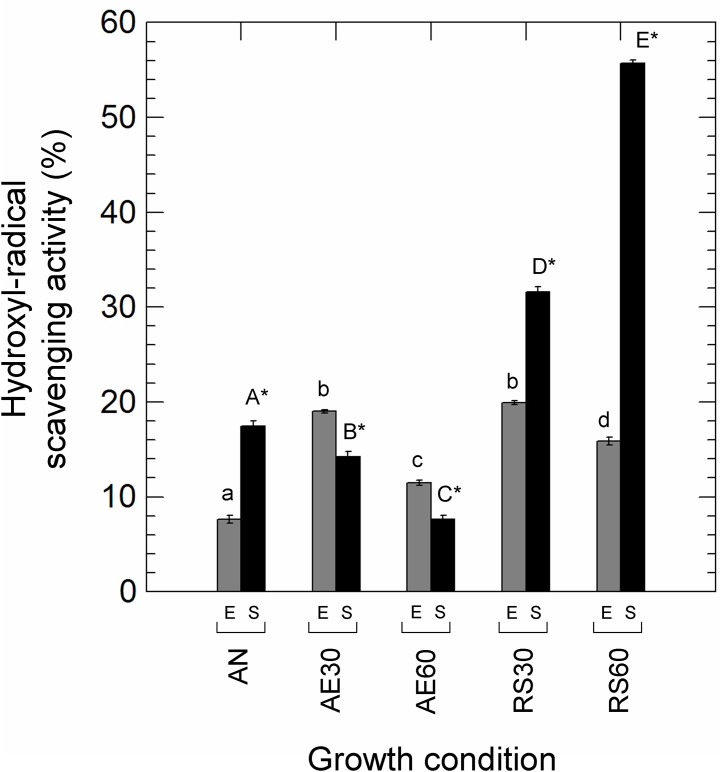
Percentage of hydroxyl radical scavenging activity in *Lactobacillus casei* N87 cultivated in different conditions. AN: anaerobic growth; AE30: aerobic growth with 30% dissolved oxygen, DO; AE60: aerobic growth with 60% DO; RS30: respiratory growth (supplementation with 2.5 μg/ml hemin and 1 μg/ml menaquinone) with 30% DO; RS60: respiratory growth (supplementation with 2.5 μg/ml hemin and 1 μg/ml menaquinone) with 60% DO. E: exponential growth phase (5 h, grey bars); S: late stationary growth phase (24 h, black bars). Lowercase letters on the bars indicate significant differences (Tukey’s HSD, *p*< 0.01) in radical scavenging activity among different growth conditions (AN, AE30, AE60, RS30, RS60) in exponential growth phase; uppercase letters on the bars indicate significant differences (Tukey’s HSD, *p*<0.01) in radical scavenging activity among different growth conditions (AN, AE30, AE60, RS30, RS60) in late stationary growth phase. * indicates significant differences (*p*<0.01) between exponential and stationary phase within the same growth condition.

The capability to remove DPPH-radicals was unaffected by growth conditions and times of incubations (ranging from 34% to 36% in all conditions), while growth conditions and growth phase strongly affected the hydroxyl radical scavenging activity (Fig 3). With the exception of unsupplemented aerated cultures (with 30% and 60% DO), the stationary phase cells (24 h) of *L*. *casei* N87 had a greater degrading activity compared to those collected in exponential growth phase (5 h). Moreover, respiratory cultivation (with 30% and 60% DO) significantly (*p*<0.01) increased the capability to remove the hydroxyl free radicals.

**Fig 4:**
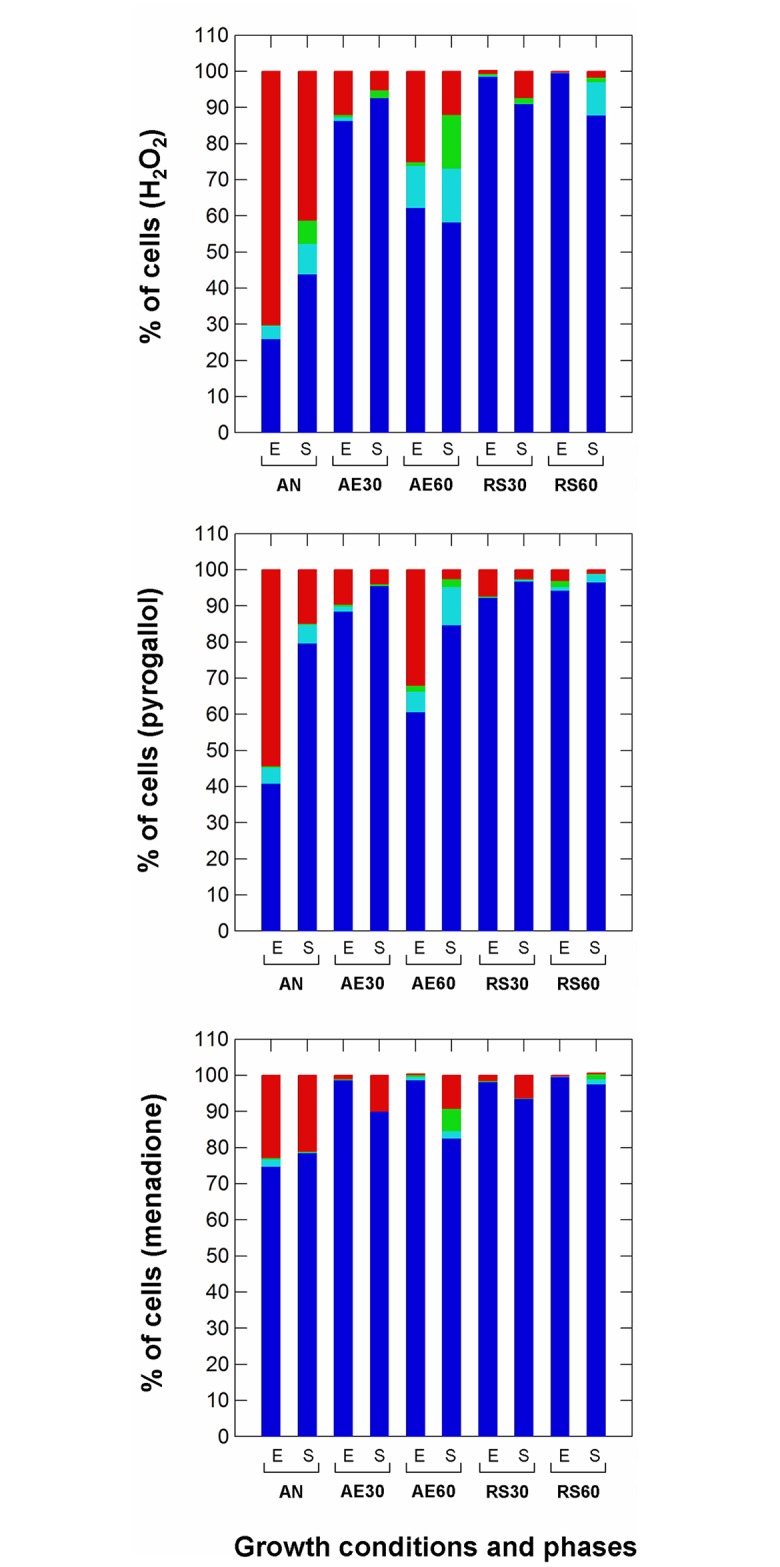
Survival after exposure to oxidative stress (hydrogen peroxide, menadione, pyrogallol) in *Lactobacillus casei* N87 cultivated in different conditions. AN: anaerobic growth; AE30: aerobic growth with 30% dissolved oxygen, DO; AE60: aerobic growth with 60% DO; RS30: respiratory growth (supplementation with 2.5 μg/ml hemin and 1 μg/ml menaquinone) with 30% DO; RS60: respiratory growth (supplementation with 2.5 μg/ml hemin and 1 μg/ml menaquinone) with 60% DO. E: exponential growth phase (5 h); S: late stationary growth phase (24 h). Blue bars: % of cultivable healthy cells (number of cultivable cells recovered on WMA68); light blue bars: % of VBNC cells (calculated as difference between the number of cultivable cells on WMAC and the number of cultivable cells on WMA68); green bars: % of damaged cells (calculated as difference between the number of cultivable cells on WMA68 and the number of cultivable cells on WMA55); red bars: % of dead cells (calculated as difference between the number of cultivable non-stressed cells and cultivable healthy+VBNC cells).

Tolerance of oxidative stress was tested using H_2_O_2_, menadione and pyrogallol (Fig 4). The latter compounds are common generators of superoxide anions. The three media used for the recovery of survivors allowed the estimations of different fractions of the surviving populations. The highest counts were consistently obtained on WMA pH 6.8, containing cysteine (WMAC), while the lowest counts were consistently obtained on WMA pH 5.5 without cysteine (WMA55). Therefore, it was assumed that the latter medium allowed only the recovery of undamaged cells, that WMA pH 6.8 without cysteine (WMA68) allowed the recovery of cultivable healthy cells and that WMAC allowed the recovery of a further fraction of viable cells, which were cultivable only in the presence of a reducing agent such as cysteine. The three different fractions are indicated by bars of different color in Fig 4. No significant differences were founds in colony counts on WMA68, WMB55 and WMAC, suggesting that the non-stressed cultures (control samples, 8 log cfu/ml) had 100% of cultivable healthy cells. The total number of survivors after either H_2_O_2_ or pyrogallol treatment was lowest for exponential cells cultivated in anaerobiosis. In addition, when cells grown in the same culture conditions were compared, the total number of survivors was always higher for stationary phase cells. In the presence of H_2_O_2_, the highest numbers of survivors (from 98% to 99% of cultivable healthy cells; blue bars in Fig 4) were measured in exponential respiratory (RS30, RS60) cultures while aerobic growth at 60% DO (AE60) apparently impaired the ability to survive both H_2_O_2_ and pyrogallol stresses compared to the other aerobic conditions. A fraction of survivors was apparently unable to grow on media at low pH and without cysteine. This fraction generally increased in stationary cells exposed to H_2_O_2_ stress. Anaerobiosis, higher-aerated (60% DO) aerobiosis (AE60) and respiration (RS60) increased the % of damaged (green bars) and viable but non cultivable (VNBC, light blue bars) cells in H_2_O_2_-stressed stationary cultures. The same behavior was found after the exposure to pyrogallol, while the tolerance of menadione was fairly high in all aerated conditions.

**Fig 5:**
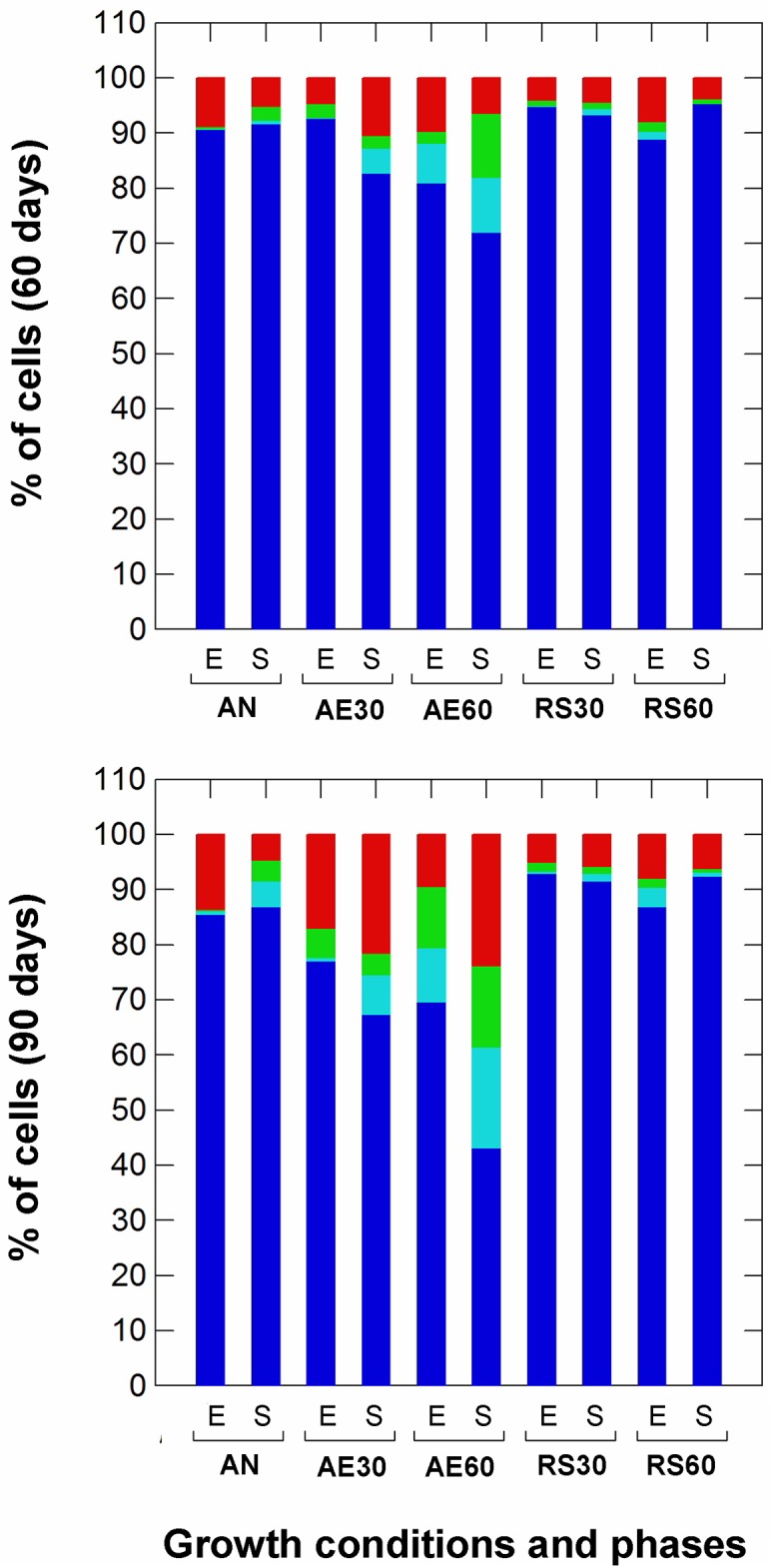
Survival of *Lactobacillus casei* N87 cultivated in different conditions to freeze-drying process, after 60 and 90 days of storage at -20°C. AN: anaerobic growth; AE30: aerobic growth with 30% dissolved oxygen, DO; AE60: aerobic growth with 60% DO; RS30: respiratory growth (supplementation with 2.5 μg/ml hemin and 1 μg/ml menaquinone) with 30% DO; RS60: respiratory growth (supplementation with 2.5 μg/ml hemin and 1 μg/ml menaquinone) with 60% DO. E: exponential growth phase (5 h); S: late stationary growth phase (24 h). Blue bars: % of cultivable healthy cells (number of cultivable cells recovered on WMA68); light blue bars: % of VBNC cells (calculated as difference between the number of cultivable cells on WMAC and the number of cultivable cells on WMA68); green bars: % of damaged cells (calculated as difference between the number of cultivable cells on WMA68 and the number of cultivable cells on WMA55); red bars: % of dead cells (calculated as difference between the number of cultivable non-stressed cells and cultivable healthy+VBNC cells).

Survival after freeze-drying was significantly (*p*<0.01) affected by the growth conditions and phase (Fig 5). High levels of DO dramatically impaired the survival (after 60 and 90 days of storage at -20°C) of unsupplemented aerated cultures (with 60% DO), leading to a higher number of VBNC and damaged cells. The aeration levels had little influence on the stress tolerance of exponential and stationary cells grown in respiratory condition with 30% DO, but affected the number of total survivors when a high oxygen level (60%) was used. However, the number of cultivable healthy cells grown under respiration was generally very high (from 89% to 95%).

## Discussion

In this work the effect of anaerobic, aerobic (O_2_) and respiratory (O_2_, heme and menaquinone) cultivation on the growth performances and stress tolerance of respiration-competent strain *L*. *casei* N87 [12] was evaluated under controlled conditions.

In contrast with results obtained by Quatravaux et al [23] on *L*. *plantarum*, lactate dehydrogenase (LDH) activity was significantly affected by aeration parameters and by the presence of respirative cofactors. During anaerobic cultivation, the activity of LDH was highest in exponential phase, when significant amounts of glucose were measured, but decreased with sugar exhaustion; the NADH-dependent reduction of pyruvate to lactate was the predominant pathway in the anaerobic cultures and lactate was the major end product at the end of fermentation. On the contrary, in lower-aerated (30% DO) and respiratory (both 30% and 60% DO) conditions, the highest levels of LDH were detected in the late exponential growth phase, when glucose concentration became limiting and when the highest activities of other flavin dependent oxidoreductases (i.e. pyruvate oxidase, POX; NADH oxidase, NOX; NADH peroxidase, NPR) were found. In presence of O_2_, in fact, the aerobic conversion of lactate into pyruvate requires NAD^+^, which in turn may be regenerated by the activity of NOX (with the reduction of O_2_ to H_2_O_2_) and/or NPR (reduction of H_2_O_2_ to water) to avoid the imbalance in NADH/NAD^+^ ratio. Contrarily to NOX and NPR (whose activities are not exclusively associated to the aerobic/respiratory metabolism; [9, 17]), POX activity was found only when O_2_ was available. However, as for the other enzymes, high concentrations (60%) of DO in unsupplemented aerated cultures significantly inhibited POX activity. Quatravaux et al [23] first investigated the effect of different DO regimes on the POX levels in *L*. *plantarum*, suggesting that 30% of DO was the optimal concentration for POX functionality. Our results confirmed these observations in unsupplemented aerobic cultures (AE30 *vs* AE60), but in respiratory conditions (RS30, RS60), the highest concentration of DO did not reduce POX activity. Probably, the activation of cytochrome *bd* oxidase (by heme addition) in the respiratory chain might reduce the levels of intracellular O_2_, avoiding its harmful effect on the enzymatic activity. Several studies, moreover, have considered others possible factors involved in the activation and regulation of POX. Lorquet et al [24] highlighted the carbon catabolite repression of *pox* gene, suggesting that the activation of POX pathway occurs in stationary growth phase when most of glucose was consumed. However, many authors [25, 9, 26] disproved these results demonstrating that at least in some strains of *L*. *plantarum* the activation and transcription of POX occurred also in the exponential growth phase. We confirmed these results for *L*. *casei* N87.

Although several authors have investigated the respiratory metabolism in LAB species, to our knowledge no study has considered the expression of the complete *cydABCD* operon (encoding for the cytochrome *bd* oxidase complex in electron transport chain in LAB) in different growth conditions and phases. *cydA* (encoding for the subunit I of cytochrome *bd* oxidase) expression has been studied by Duwat et al ([27], Northern blot analysis) and by Arioli et al ([28], qRT-PCR analysis) in *Lc*. *lactis*, and by Ianniello et al ([29], qRT-PCR analysis) in *L*. *rhamnosus* and *L*. *spicheri* under anaerobic and aerobic cultivations, with or without heme supplementation (uncontrolled shaken flask experiments). Moreover, a number of *cydA* knockout mutants have been constructed and used to confirm the implication of cytochrome *bd* oxidase in the activation of respiratory chain and O_2_ tolerance in some LAB species [27, 30]. Puri-Taneja et al [31] suggested a CcpA-mediated repression in *cydABCD* operon of *Bacillus subtilis*. To our knowledge, no transcriptional regulator has been yet recognized for the *cydABCD* operon of LAB.

We found that the transcription of *pox* in *L*. *casei* N87 was strongly regulated by the dissolved O_2_ concentration, while the presence of hemin was the main factor in the induction of cytochrome *bd* oxidase. Moreover, heme supplementation and high O_2_ levels increased, respectively, the expression of *pox* and *cydABCD* genes in respiratory cells. Our results demonstrated that the shift towards aerobic (*pox* transcription) and respiratory (*cydABCD* transcription) growth occurred during the exponential phase, indicating that aerobic respiration may be an alternative energetically favorable pathway in respiration-competent strains, and not exclusively a secondary process for growth maintenance in limited sugar conditions, as previously suggested [2, 14–15].

Several reactive oxygen species (ROS) may be produced in the presence of O_2_. In this study, the accumulation of H_2_O_2_ (an important by product of POX activity) was the highest in aerobic growing cells, while in respirative conditions was significantly lower. Low H_2_O_2_ concentration may be due to higher levels of catalase activity detected in respirative cultures. Catalase activity was also measured in anaerobic and unsupplemented aerobic cells, suggesting the presence of both heme-dependent and Mn-dependent catalases in *L*. *casei* N87. This is confirmed by genome information [21] with the presence of sequences encoding for heme-catalase (1461-bp) and Mn-catalase (936-bp). The presence of O_2_ increases the activity of both catalases [Zotta T., unpublished data]. Degradation of H_2_O_2_ in lower-aerated (AE30) and respirative (RS30, RS60) cells, moreover, may be due to the highest levels of NPR detected in above growth conditions. Respiratory growth also promoted radical scavenging activity in *L*. *casei* N87, as previously shown by Ianniello et al [13]. The capability of the respiratory phenotype to tolerate O_2_ and prevent ROS accumulation contributed to increased survival to oxidative and freeze-drying stresses. Respiratory cells of *L*. *casei* N87, in fact, showed a greater stress tolerance than those grown in anaerobiosis or unsupplemented aerobiosis, while the accumulation of O_2_ and ROS in higher-aerated unsupplemented cultures (AE60) resulted in an increase in VBNC and damaged cells, confirming the noxious effect of O_2_-related compounds, which may cause sub-lethal damage in addition to cell death. The robustness to oxidative stress and storage was previously demonstrated in respirative phenotypes of *L*. *plantarum* [32, 9–10] and *Lc*. *lactis* ([33, 34], confirming that respiration may be a useful strategy to reduce oxidative damage in some LAB species. This is also true in other bacteria. In fact, several authors have found that in *Escherichia coli* the cytochrome *bd* oxidase, as well H_2_O_2_-degranding activity [35], may increase the tolerance of nitric oxide [36] and sulphides [37], suggesting that aerobic respiration may accomplish either bioenergetic functions and stress adaptation.

## Conclusion

Respiration might be successfully exploited to develop natural boosted and food-grade phenotypes (without DNA manipulation). Respiratory growth, in fact, may improve biomass production and features stress tolerance of *L*. *casei* N87 compared to anaerobic and unsupplemented aerobic cultivations. Specifically, the activation of respiratory pathways (synthesis of cytochrome *bd* oxidase, utilization of O_2_ as electron acceptor in ET chain) may prevent the accumulation of O_2_ and toxic radicals that may negatively affect the enzymatic activities, stress resistance and antioxidant capability of *L*. *casei* N87.

This work, moreover, offers progress in the knowledge on the regulation of aerobic and respiratory growth of *L*. *casei*, and generally of LAB, since for the first time the gene expression of *pox* and *cydABCD* operon was evaluated in different growth conditions (anaerobiosis *vs* aerobiosis *vs* respiration), phases (from early exponential to late stationary) and aeration parameters (30% and 60% of DO). However, a greater understanding of gene regulation and metabolic pathways of anaerobic and respirative phenotypes may be useful to control and manipulate the fitness of respiration-competent strains and to exploit them in different food (production of aroma compounds and reduction of oxidative processes in fermented foods) and health (removal of toxic compounds) related applications.

## Supporting Information

S1 Fig Kinetics of growth of *Lactobacillus casei* N87. Circles: anaerobic growth; up-triangles: aerobic growth with 30% dissolved oxygen, DO; low-triangles: aerobic growth with 60% DO; left-triangles: respiratory growth with 30% DO; squares: respiratory growth with 60% DO. Continuous lines show the fit of the Baranyi and Roberts model [18].
(TIF)

S2 Fig Correlation between enzymatic activity of pyruvate oxidase (POX) and its relative gene expression (*pox*) in *Lactobacillus casei* N87. Circles: anaerobic growth; up-triangles: aerobic growth with 30% dissolved oxygen, DO; low-triangles: aerobic growth with 60% DO; left-triangles: respiratory growth with 30% DO; squares: respiratory growth with 60% DO. White symbols: cultures at 5 h of incubation; grey symbols: cultures at 7 h of incubation; black symbols: cultures at 24 h of incubation.
(TIF)

S1 Table Sequences of forward (F) and reverse (R) primers used for quantitative Real Time PCR. (DOCX)

## References

[1] Lechardeur D, Cesselin B, Fernandez A, Lamberet G, Garrigues C, Pedersen M, et al Using heme as an energy boost for lactic acid bacteria. Curr Opin Biotech. 2011; 22: 143–149. 10.1016/j.copbio.2010.12.001 21211959

[2] Pedersen MB, Gaudu P, Lechardeur D, Petit MA, Gruss A. Aerobic respiration metabolism in lactic acid bacteria and uses in biotechnology. Annu Rev Food Sci Technol. 2012; 3: 37–58. 10.1146/annurev-food-022811-101255 22385163

[3] Brooijmans RJW, Smit B, Santos F, van Riel J, de Vos WM, Hugenholtz J. Heme and menaquinone induced electron transport in lactic acid bacteria. Microbial Cell Fact. 2009a; 8: 28 10.1186/1475-2859-8-28 19480672 PMC2696406

[4] Borisov VB, Gennis RB, Hemp J, Verkhovsky MI. The cytochrome bd respiratory oxygen reductases. Biochim Biophys Acta. 2011a; 1807: 1398–1413. 10.1016/j.bbabio.2011.06.016 21756872 PMC3171616

[5] Borisov VB, Muralib R, Verkhovskaya ML, Bloch DA, Hanb H, Gennis RB, et al Aerobic respiratory chain of *Escherichia coli* is not allowed to work in fully uncoupled mode. PNAS. 2011b; 108: 17320–17324. 10.1073/pnas.1108217108 21987791 PMC3198357

[6] Pedersen MB, Iversen SL, Sorensen KI, Johansen E. The long and winding road from the research laboratory to industrial applications of lactic acid bacteria. FEMS Microbiol Rev. 2005; 29: 611–624. 10.1016/j.femsre.2005.04.001 15935510

[7] Brooijmans RJW, de Vos WM, Hugenholtz J. *Lactobacillus plantarum* electron transport chain. Appl Environ Microbiol. 2009b; 75: 3580–3585. 10.1128/AEM.00147-09 19346351 PMC2687314

[8] Guidone A, Ianniello RG, Ricciardi A, Zotta T, Parente E. Aerobic metabolism and oxidative stress tolerance in the *Lactobacillus plantarum* group. Word J Microbiol Biotechnol. 2013; 29: 1713–1722. 10.1007/s11274-013-1334-0 23543191

[9] Zotta T, Guidone A, Ianniello RG, Parente E, Ricciardi A. Temperature and respiration affect the growth and stress resistance of *Lactobacillus plantarum* C17. J Appl Microbiol. 2013; 115: 848–858. 10.1111/jam.12285 23782242

[10] Zotta T, Ianniello RG, Guidone A, Parente E, Ricciardi A. Selection of mutants tolerant of oxidative stress from respiratory cultures of *Lactobacillus plantarum* C17. J Appl Microbiol. 2014a; 116: 632–643. 10.1111/jam.12398 24267916

[11] Ricciardi A, Ianniello RG, Tramutola A, Parente E, Zotta T. Rapid detection assay for oxygen consumption in the *Lactobacillus casei* group. Ann Microbiol. 2014; 64: 1861–1864. 10.1007/s13213-014-0819-x

[12] Zotta T, Ricciardi A, Ianniello RG, Parente E, Reale A, Rossi F, et al Assessment of aerobic and respiratory growth in the *Lactobacillus casei* group. PLoS ONE. 2014b; 9: e99189 10.1371/journal.pone.0099189 24918811 PMC4053349

[13] Ianniello RG, Ricciardi A, Parente E, Tramutola A, Reale A, Zotta T. Aeration and supplementation with heme and menaquinone affect survival to stresses and antioxidant capability of *Lactobacillus casei* strains. LWT-Food Sci Technol. 2015a; 60: 817–824. 10.1016/j.lwt.2014.10.020

[14] Lopez de Felipe F, Gaudu P. Multiple control of the acetate pathway in *Lactococcus lactis* under aeration by catabolite repression and metabolites. Appl Microbiol Biotechnol. 2009; 82: 1115–1122. 10.1007/s00253-009-1897-8 19214497

[15] Goffin P, Muscariello L, Lorquet F, Stukkens A, Prozzi D, Sacco M, et al Involvement of pyruvate oxidase activity and acetate production in the survival of *Lactobacillus plantarum* during the stationary phase of aerobic growth. Appl Environ Microbiol. 2006; 72: 7933–7940. 10.1128/AEM.00659-06 17012588 PMC1694206

[16] Iacumin L, Ginaldi F, Manzano M, Anastasi V, Reale A, Zotta T, et al High resolution melting analysis (HRM) as a new tool for the identification of species belonging to the *Lactobacillus casei* group and comparison with species-specific PCRs and multiplex PCR. Food Microbiol. 2015; 46: 357–67. 10.1016/j.fm.2014.08.007 25475306

[17] Zotta T, Ricciardi A, Guidone A, Sacco M, Muscariello L, Mazzeo MF. Inactivation of ccpA and aeration affect growth, metabolite production and stress tolerance of *Lactobacillus plantarum* WCFS1. Int J Food Microbiol. 2012; 155: 51–59. 10.1016/j.ijfoodmicro.2012.01.017 22326142

[18] Baranyi J, Roberts TA. A dynamic approach to predicting bacterial growth in food. Int J Food Microbiol. 1994; 23: 277–294. 10.1016/0168-1605(94)90157-0 7873331

[19] Baranyi J, Le Marc Y. Dmfit Manual, Version 2.0. Norwich, UK: Institute of Food Research; 1996.

[20] Wang AN, Yi XW, Yu HF, Dong B, Qiao SY. Free radical scavenging activity of *Lactobacillus fermentum* in vitro and its antioxidative effect on growing-finishing pigs. J App Microbiol. 2009; 107: 1140–1148. 10.1016/j.lwt.2014.10.020 19486423

[21] Zotta T, Ricciardi A, Parente E, Reale A, Ianniello RG, Bassi D. Draft genome sequence of the respiration-competent strain *Lactobacillus casei* N87. Genome Announc. 2016; 4: e00348–16. 10.1128/genomeA.00348-16 27151805 PMC4859187

[22] Pfaffl MW. A new mathematical model for relative quantification in real-time RT-PCR. Nucleic Acids Res. 2001; 29: e45 10.1093/nar/29.9.e45 11328886 PMC55695

[23] Quatravaux S, Remize F, Bryckaert E, Colavizza D, Guzzo J. Examination of *Lactobacillus plantarum* lactate metabolism side effects in relation to the modulation of aeration parameters. J Appl Microbiol. 2006; 101: 903–912. 10.1111/j.1365-2672.2006.02955.x 16968302

[24] Lorquet F, Goffin P, Muscariello L, Baudry JB, Ladero V, Sacco M et al Characterization and functional analysis of the *poxB* gene, which encodes pyruvate oxidase in *Lactobacillus plantarum*. J Bacteriol. 2004; 186: 3749–3759. 10.1128/JB.186.12.3749-3759.2004 15175288 PMC419957

[25] Stevens MJA, Wiersma A, de Vos WM, Kuipers OP, Smid EJ, Molenaar D, et al Improvement of *Lactobacillus plantarum* aerobic growth as directed by comprehensive transcriptome analysis. Appl Environ Microbiol. 2008; 74: 4776–4778. 10.1128/AEM.00136-08 18539801 PMC2519335

[26] Ricciardi A, Castiglione Morelli MA, Ianniello RG, Parente E, Zotta T. Metabolic profiling and stress response of anaerobic and respiratory cultures of *Lactobacillus plantarum* C17 grown in a chemically defined medium. Ann Microbiol. 2015; 65: 1639–1648. 10.1007/s13213-014-1003-z

[27] Duwat P, Sourice S, Cesselin B, Lamberet G, Vido K, Gaudu P. et al Respiration capacity of the fermenting bacterium *Lactococcus lactis* and its positive effects on growth and survival. J Bacteriol. 2001; 183: 4509–4516. 10.1128/JB.183.15.4509-4516.2001 11443085 PMC95345

[28] Arioli S, Zambelli D, Guglielmetti S, De Noni I, Pedersen MB, Pedersen PD, et al Increasing the heme-dependent respiratory efficiency of *Lactococcus lactis* by inhibition of lactate dehydrogenase. Appl Environ Microbiol. 2013; 79: 376–80. 10.1128/AEM.02734-12 23064338 PMC3536120

[29] Ianniello RG, Zheng J, Zotta T, Ricciardi A, Gänzle MG. Biochemical analysis of respiratory metabolism in the heterofermentative *Lactobacillus spicheri* and *Lactobacillus reuteri*. J Appl Microbiol. 2015b; 119: 763–775. 10.1111/jam.12853 25996113

[30] Brooijmans R, Poolman B, Schuurman-Wolters GK, de Vos WM, Hugenholts J. Generation of a membrane potential by *Lactococcus lactis* through aerobic electron transport. J Bacteriol. 2007; 189: 5203–5209. 10.1128/JB.00361-07 17496098 PMC1951855

[31] Puri-Taneja A, Schau M, Chen M, Hulett FM. Regulators of the *Bacillus subtilis cydABCD* operon: identification of a negative regulator, CcpA, and a positive regulator, ResD. J Bacteriol. 2007; 189: 3348–3358. 10.1128/JB.00050-07 17322317 PMC1855890

[32] Watanabe M, van der Veen S, Nakajima H, Abee T. Effect of respiration and manganese on oxidative stress resistance of *Lactobacillus plantarum* WCFS1. Microbiol. 2012; 158: 293–300. 10.1099/mic.0.051250-0 22016573

[33] Rezaïki L, Cesselin B, Yamamoto Y, Vido K, van West E, Gaudu P, et al Respiration metabolism reduces oxidative and acid stress to improve long-term survival of *Lactococcus lactis*. Mol Microbiol. 2004; 53: 1331–1342. 10.1111/j.1365-2958.2004.04217.x 15387813

[34] Cesselin B, Derré-Bobillot A, Fernandez A, Lamberet G, Lechardeur D, Yamamoto Y, et al Respiration, a strategy to avoid oxidative stress in *Lactococcus lactis*, is regulated by the heme status. Jpn J Lactic Acid Bact. 2010; 21: 10–15. 10.4109/jslab.21.10

[35] Borisov VB, Forte E, Davletshin A, Mastronicola D, Sarti P, Giuffrè A. Cytochrome bd oxidase from *Escherichia coli* displays high catalase activity: An additional defence against oxidative stress. FEBS Lett. 2013; 587: 2214–2218. 10.1016/j.febslet.2013.05.047 23727202

[36] Giuffrè A, Borisov VB, Mastronicola D, Sarti P, Forte E. Cytochrome bd oxidase and nitric oxide: from reaction mechanisms to bacterial physiology. FEBS Lett. 2012; 586: 622–629. 10.1016/j.febslet.2011.07.035 21821033

[37] Forte E, Borisov VB, Falabella M, Colaço HG, Tinajero-Trejo M., Poole RK, et al The terminal oxidase cytochrome bd promotes sulfide-resistant bacterial respiration and growth. Sci Rep. 2016; 6: 23788 10.1038/srep23788 27030302 PMC4815019

