# Supplementary figures and images for: Investigation of Factors Affecting Aerobic and Respiratory Growth in the Oxygen-Tolerant Strain Lactobacillus casei N87
Source: PLoS One. 2016 Nov 3;11(11):e0164065. doi: 10.1371/journal.pone.0164065 (PMC5094797; doi:10.1371/journal.pone.0164065)

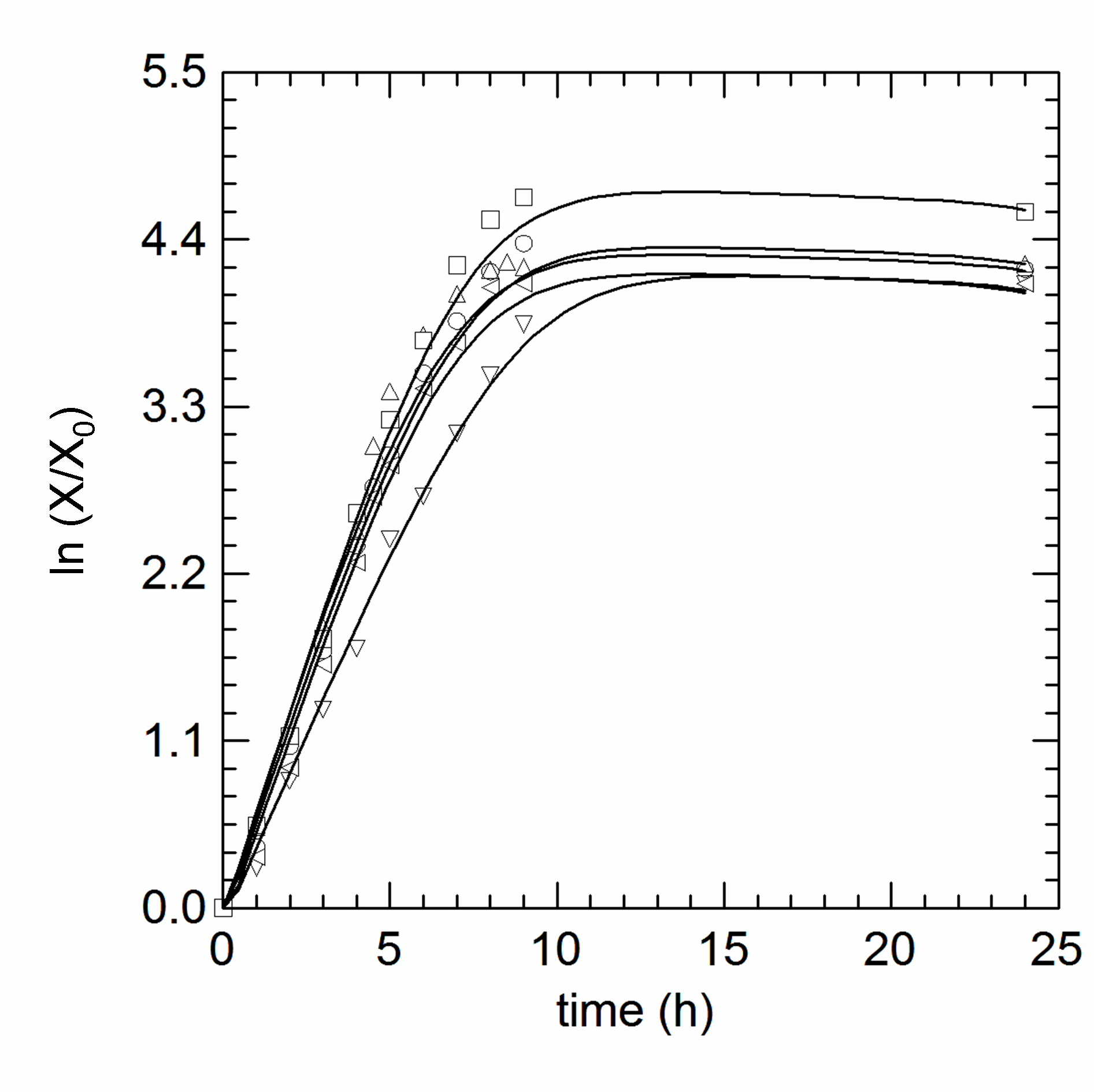

Supplement: S1 Fig — Circles: anaerobic growth; up-triangles: aerobic growth with 30% dissolved oxygen, DO; low-triangles: aerobic growth with 60% DO; left-triangles: respiratory growth with 30% DO; squares: respiratory growth with 60% DO. Continuous lines show the fit of the Baranyi and Roberts model [18]. (TIF) [file pone.0164065.s001.tif]

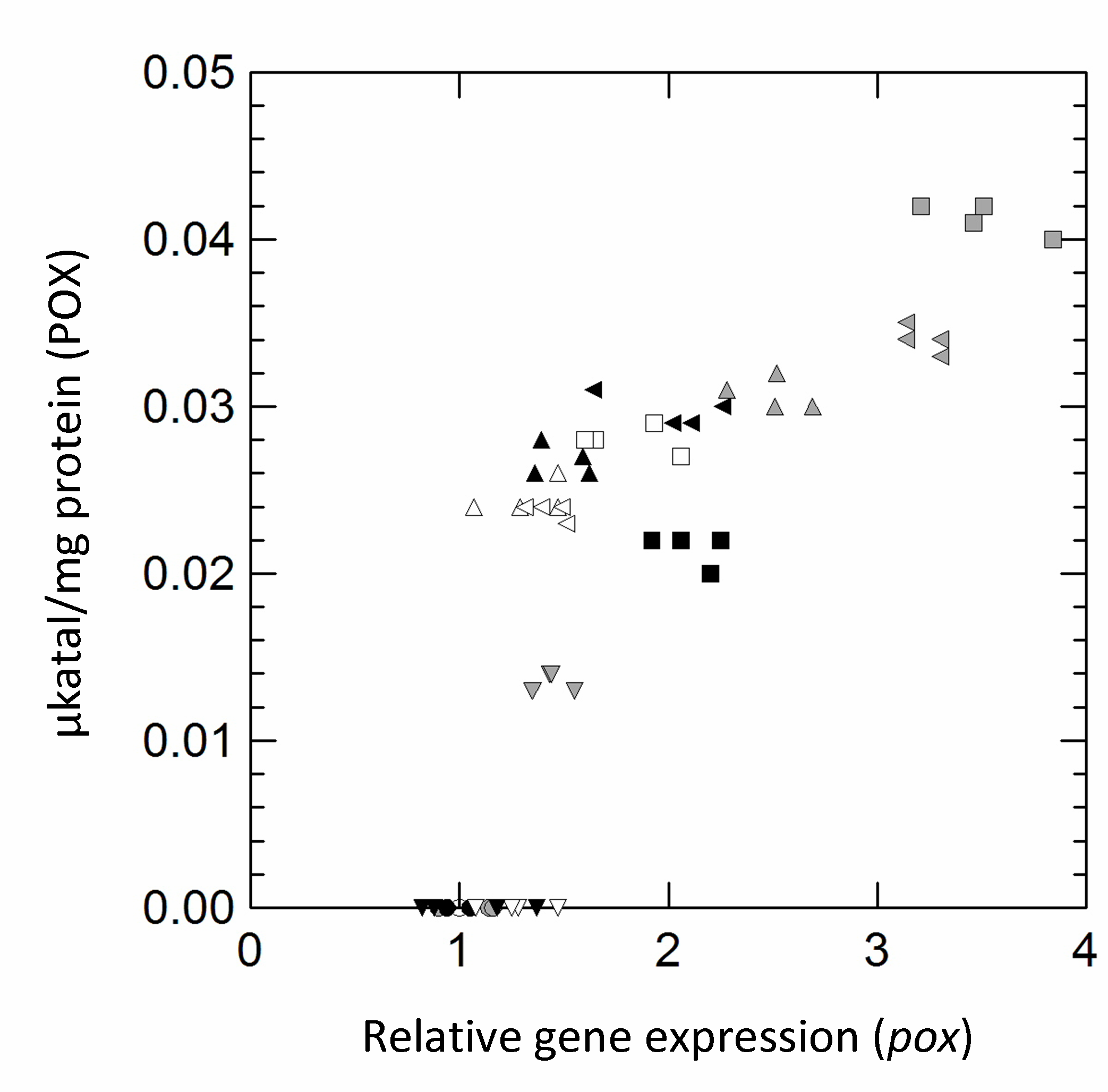

Supplement: S2 Fig — Circles: anaerobic growth; up-triangles: aerobic growth with 30% dissolved oxygen, DO; low-triangles: aerobic growth with 60% DO; left-triangles: respiratory growth with 30% DO; squares: respiratory growth with 60% DO. White symbols: cultures at 5 h of incubation; grey symbols: cultures at 7 h of incubation; black symbols: cultures at 24 h of incubation. (TIF) [file pone.0164065.s002.tif]
