# Supplementary material for: Investigation of Factors Affecting Aerobic and Respiratory Growth in the Oxygen-Tolerant Strain Lactobacillus casei N87
Source: PLoS One. 2016 Nov 3;11(11):e0164065. doi: 10.1371/journal.pone.0164065 (PMC5094797; doi:10.1371/journal.pone.0164065)
Supplement: S1 Table — (DOCX) [file pone.0164065.s003.docx]

**S1 Table. Sequences of forward (F) and reverse (R) primers used for quantitative Real Time PCR.**

| **Gene ^a^** | **Primer name** | **Sequence** |
| --- | --- | --- |
| *gadph* | gadph-N87-F | 5’-GCACACCGAAAACAACCCTAGC-3’ |
| *gadph* | gadph-N87-R | 5’-CGTTATCGTACCAAGCAACCGT-3’ |
| *gyrB* | gyrB-N87-F | 5’-CGCTGCTGACGTTTGCCAAT-3’ |
| *gyrB* | gyrB-N87-R | 5’-TGACCGAGACAACTGCCGTT-3’ |
| *tuf* | tuf-N87-F | 5’-CCACACCACCGATGTTACCG-3’ |
| *tuf* | tuf-N87-R | 5’-ACAGTACGACCGCCTTCACG-3’ |
| *cydA* | cydA-N87-F | 5’-AGGATCACACGACCAAAGCCA-3’ |
| *cydA* | cydA-N87-R | 5’-GCATCGTCCAGAACAGCAGC-3’ |
| *cydB* | cydB-N87-F | 5’-GGCGGTGCGATGTTTGCTT-3’ |
| *cydB* | cydB-N87-R | 5’-GACAAACGGCGGAATCAGGC-3’ |
| *cydC* | cydC-N87-F | 5’-TTGTCCTGACCCGTGCCTTT-3’ |
| *cydC* | cydC-N87-R | 5’-GTTCCGAGACAAATGCCGCA-3’ |
| *cydD* | cydD-N87-F | 5’-GAACCAAGGTGCCGCATCTG-3’ |
| *cydD* | cydD-N87-R | 5’-CAGTGACTCAAACCTGCCGC-3’ |
| *pox* | pox-N87-F | 5’-CGACTGCTGCCATGAACACC-3’ |
| *pox* | pox-N87-R | 5’-GAATCGTCACCACCGCTACG-3 |

**^a^** *gapdh*, glyceraldehyde-3-phosphate dehydrogenase; *gyrB*, DNA gyrase subunit B; *tuf*, elongation factor Tu;

*cydA*, subunit I of cytochrome D ubiquinol oxidase; *cydB*, subunit II of cytochrome D ubiquinol oxidase;

*cydC*, ABC transporters of cytochrome D subunit III; *cydD*, ABC transporters of cytochrome D subunit IV;

*pox*, pyruvate oxidase.
